# Magnitude of HIV Infection and Associated Factors among Female Sex Workers at Hawassa, Ethiopia

**DOI:** 10.4314/ejhs.v32i2.6

**Published:** 2022-03

**Authors:** Getahun Hilemeskel Alemu, Deresse Daka Gidebo, Musa Mohammed Ali

**Affiliations:** 1 Hawassa University Comprehensive Specialized Hospital, Hawassa, Ethiopia; 2 Hawassa University, College of Medicine and Health Sciences, School of Medical Laboratory Science, Hawassa, Ethiopia

**Keywords:** Magnitude of HIV, Female sex workers, Risk factors, Hawassa, Ethiopia

## Abstract

**Background:**

HIV is among one of the most serious public health problems. Low-income countries are highly affected by Human Immuno-deficiency Virus (HIV). The burden of HIV varies across various segments of the population. The aim of this study was to determine the magnitude of HIV infection and associated factors among female sex workers (FSWs).

**Methods:**

A cross-sectional study was conducted among 381 FSWs at Integrated Service on Health and Development Organization (ISHDO) located in Hawassa city from July to November 2018. Socio-demographic and related data were collected using a structured questionnaire. About 5 ml of venous blood was collected from study participants; serum was prepared and tested for HIV using the 4^th^ generation Microlisa HIV assay. Data were analyzed by using SPSS version 21; binary and multivariable logistic regressions were used to determine factors associated with HIV infection among FSWs. A p-value of less than 0.05 was considered statistically significant.

**Results:**

The prevalence of HIV among FSWs at ISHDO, Hawassa was 19.9% [95% CI: 16, 24.4].

**Conclusion:**

The prevalence of HIV among FSWs at ISHDO, Hawassa was relatively high compared to national and regional reports. In this study, none of the factors assessed were significantly associated with HIV infection.

## Introduction

In spite of the global efforts to reduce Human Immuno-deficiency Virus (HIV) infections, it remains one of the most important public health problems. Until now, over 70 million individuals have been infected and about 30 million individuals have died as a result of HIV-related illnesses. At the end of 2019, 38 million people were living with HIV globally (1). The burden of HIV infection varies from country to country, within the country, and across different segments of the population while the African region remains most severely affected (1).

In Ethiopia, in 2018, 690,000 people were living with HIV and the prevalence of HIV among adults was 1%, which is greater than the global report. In 2018, about 23, 000 people were newly infected with HIV and 11000 people died of AIDS-related illnesses (2).

Certain segments of the population such as female sex workers (FSWs), who exchange sexual services for money, are disproportionately affected by HIV compared to the general female population. Structural factors that predispose FSWs to HIV infection include work environment, poverty, stigma, and discrimination (3). Behavioral factors which predispose to HIV infection include a large number of sex partners, intermittent use of condoms; engaging in high-risk sex, drug use, and co-infection with other sexually transmitted diseases (4, 5). In Ethiopia, sex work for a living is not legalized. However, FSWs are mostly based in legally recognized establishments such as hotels and bars. FSWs are exposed to many challenges: among them are sexually transmitted infections, including HIV. Limited data from Ethiopia indicated that the prevalence of HIV among FSWs to be varied between 20% and 75% (6, 7). FSWs in Ethiopia are not formally organized like FSWs in other countries, this by itself makes them more prone to HIV infection.

Globally, UNAIDS estimates that less than 50% of FSWs have access to HIV prevention programs (8). With very few structural interventions and rare organizations on the ground, FSWs in most parts of Africa remain highly exposed to HIV infection. Moreover, FSWs in Africa are in extreme poverty and they do not get appropriate support from family and society.

Since Female Sex Workers (FSWs) are highly affected by HIV they can be a source of infection for their clients and to the community at large. Even though the prevalence of HIV is declining among the general population, high-risk groups still require attention to strengthen the prevention and control of HIV infection. So far there are few formally structured studies to address the magnitude of HIV among FSWs in Hawassa. In this study, we aimed to measure the magnitude and associated factors of HIV infection among FSWs attending Integrated Service on Health and Development Organization (ISHDO) in Hawassa city, Ethiopia.

## Methods

**Study area**: This study was conducted at integrated service on health and development organization (ISHDO) project confidential clinic found in Hawassa city, Sidama Regional State. It is one of the non-governmental institutions which provide support for health-related services for the marginalized populations. Hawassa is located on the shores of Lake Hawassa in the Great Rift Valley, about 275 km to the South of Addis Ababa, the Capital city of Ethiopia. The city has eight sub-cities and a total population of 302,000 according to the 2021 estimate.

**Study design and period**: A cross-sectional study was conducted from July to November 2018

**Dependent variable**: HIV test result

**Independent variables**: Socio-demographic and other factors that could predispose to HIV infections

**Study population and sample size determination**: FSWs that live in Hawassa city and whose age is greater than 16 years were included in the study. The sample size was determined using single proportion formula by considering 50% prevalence of HIV, with 95% confidence interval, 5% margin of error, considering risk factors, use of correction formula (since the total number of FSWs were less than 10,000). Based on the above, the total sample size was 383. To recruit participants, a systematic random sampling technique was used; the first participant was selected by using lottery methods.

**Data collection**: Socio-demographic characteristics and factors associated with HIV infection were collected from consented and consulted FSWs by using the Amharic version of a structured questionnaire. From all study participants, about 5 ml of venous blood was collected in test tubes containing anticoagulant following aseptic technique, and serum was prepared by centrifuging blood at 5000 revolution/minute for 10 minutes. Infection with HIV was determined by using the 4^th^ generation Microlisa HIV assay (Indiamart, India). Briefly, specimens and controls were added to the microtiter wells and incubated. The plate is then washed to remove unbound material. Horseradish peroxidase conjugated gp41, C-terminus of gp120 of HIV-1, and gp36 of HIV-2 and anti-p24 antibodies were added to each well. Substrate solution containing chromogen and hydrogen peroxide was added to the wells and incubated. The color reaction is stopped by a stop solution. The enzyme substrate reaction was read by the Enzyme Immuno Assay reader for absorbance at a wavelength of 450 nm. If the sample did not contain HIV-1 or HIV-2 antibodies or HIV-1 p24 antigen, then enzyme conjugate will not bind and the solution in the wells will be either colorless or only a faint background color develops.

To maintain the quality of data, the questionnaire which was originally prepared in English was translated to Amharic and then translated back to English. The questionnaire was pretested on 5% of the total sample size. Captured data was reviewed and checked every day for completeness. Laboratory procurers were conducted according to the manufacturer's manual. Prior to the laboratory test, the method was checked by using external known positive and negative controls.

**Data analysis**: Data were analyzed by using SPSS version 21 software; results were summarized and presented in text and tables. Binary and multivariable logistic regressions were used to determine factors associated with HIV infection. Factors with a P-value less than 0.025 were selected for further analysis by multivariable logistic regression. A P-value of less than 0.05 was considered statistically significant.

**Ethical Approval**: The research has been ethically cleared by Hawassa University College of Medicine and Health Sciences institutional review board (IRB). Permission had been guaranteed from the clinics. Clients participated after informed consent was obtained and voluntarily after they were informed about the research's risk or benefits. They were informed not to participate or might leave the study at any time. Their decision not to participate in the study did not affect their benefits to which they were informed about the study. Confidentiality was kept by using codes instead of names that could relate to the participants.

## Results

**Socio-demographic characteristics of study participants**: In the current study, out of 383 FSWs approached, 381 were recruited. The majority of study participants fell in the age category of 20–24 years (45.6%), most were not married (75.9%) and 80.3% of them attended some formal education (Table 1). 93.3% of participants used condoms of which 82.6% used them consistently during sexual intercourse. 65.3% of participants engaged in sex work for 2 to 5 years; 25.7% had a history of sexually transmitted infection (STI); 45.2% of participants work at hotels (Table 2).

**Table 1:** Socio-demographic characteristics of female sex workers attending Integrated Service on Health and Development Organization (ISHDO), Hawassa, Ethiopia, 2018 (N=381)

Socio-demographic characteristics		n (%)
Age in years	15–19	91 (23.9)
	20–24	174 (45.6)
	25–29	90 (23.6)
	30–34	17 (4.5)
	35–40	9 (2.4)
Marital status	Married	11 (2.9)
	Not married	289 (75.9)
	Divorced	37 (9.7)
	Widowed	44 (11.5)
Educational status	No formal education	75 (19.7)
	Primary school	264 (69.3)
	High school and above	42 (11)
Monthly income in Ethiopian birr	501–1000	104 (27.3)
	1001–1500	87 (22.8)
	1501–2000	130 (34.1)
	2001–2500	50 (13.2)
	>2500	10 (2.6)
Depend on family for living	Yes	295 (77.4)
	No	86 (22.6)
Dependants to live with	Yes	157 (41.2)
	No	224 (58.8)

HIV: Human Immuno deficiency Virus, n: number

**Table 2:** Frequency of factors that could be associated with HIV infection among female sex workers attending Integrated Service on Health and Development Organization (ISHDO), Hawassa, Ethiopia, 2018 (N=381)

Factors that could predispose to HIV infection		n (%)
Use of Condom	Yes	367 (93.3)
	No	14 (3.7)
Frequency of condom use	Always	303 (82.6)
	Some times	53 (14.4)
	Rarely	11 (3.0)
Breakage of condom	Yes	103 (28.1)
	No	264 (71.9)
Alcohol consumption	Yes	318 (83.5)
	No	63 (16.5)
Year of work as sex worker	<1	65 (17.1)
	2–5	249 (65.3)
	>5	67 (17.6)
Types of sex	Vaginal only	287 (75.3)
	Vaginal and anal	39 (10.2)
	Vagina and oral	55 (14.5)
History of STI	Yes	94 (24.7)
	No	287 (75.3)
History of genital ulcer	Yes	99 (25.9)
	No	282 (74.1)
Numbers of clients served per day	1–2	224 (58.8)
	3–6	157 (41.2)
Sex during menses	Yes	11(2.9)
	No	370(97.1)
Sexual assault	Yes	44 (11.5)
	No	337 (88.5)
History of abortion	Yes	36 (9.5)
	No	345 (90.5)
Place of abortion	Heath facility	11 (30.6)
	Not health facility	25 (69.4)
Place of sex	Hotel	172 (45.2)
	Street	160 (41.9)
	Home	49 (12.9)

HIV: Human Immuno deficiency Virus, n: number, STI: Sexual transmitted infection

**Prevalence of HIV among female sex workers**: From 381 participants, 76(19.9%) 95% [CI: 16, 24.4] were positive for HIV. Among the factors we assessed, none of them were significantly associated with HIV infection in multivariate logistic regression analysis (P>0.05); however, the prevalence of HIV was higher among participants within the age group of 15–19 years (24.2%), widowed (27.3%), those with a monthly income of 501–1000 (26.9%), and those who don't use condoms (21.4%) (Table 3).

**Table 3:** Bivariate and multivariate analysis of factors that could be predispose to HIV infection among female sex workers attending Integrated Service on Health and Development Organization (ISHDO), Hawassa, Ethiopia, 2018 (N=381)

Variables	Total n (%)	HIV test result					

		Positive n (%)	Negative n (%)	COR (95% CI)	P- value	AOR (95% CI)	P- value
**Age category in years**							
15–19	91	22(24.2)	69(75.8)	2.5(0.62–10.2)	0.12	0.49(0.11–2.23)	0.37
20–24	174	31(17.8)	143(82.2)	3.7(0.94–14.5)	0.06	0.33(0.08–1.41)	0.13
25–29	90	16(17.8)	74(82.2)	3.7(0.89–15-3)	0.07	0.35(0.08–1.61)	0.18
30–34	17	3(17.6)	14(82.4)	3.7(0.61–22.9)	0.15	0.34(0.05–2.24)	0.26
35–40	9	4(44.4)	5(55.6)	1			
**Use of condom**							
No	14	3(21.4)	11(78.6)	0.91(0.25–3.35)	0.88		
Yes	367	73(19.9)	294(80.1)	1			
**Breakage of condom**							
Yes	103	17(16.5)	86(83.5)	1.4(0.77–2.55)	0.26	0.68(0.37–1.25)	0.22
No	264	56(21.2)	208(78.8)	1			
**Sex during menstrual cycle**							
Yes	11	4(36.4)	7(63.6)	2.37(0.67–8.29)	0.18	2.5(0.69–9.06)	0.16
No	370	72(19.5)	298(80.5)	1			
**Sexual assault**							
Yes	44	12(27.3)	32(72.7)	1.6(0.78–3.28)	0.19	1.59(0.76–3.33)	0.22
No	337	64(19)	273(81)	1			
**Place of sex**							
Hotel	172	28(16.3)	144(83.7)	2.27(1.09–4.71)	0.028	0.51(0.24–1.11)	**0.089**
Street	160	33(20.6)	127(79.4)	1.69(0.83–3.48)	0.15	0.63(0.29–1.37)	0.25
Home	49	15(30.6)	34(69.4)	1			

HIV: Human Immuno deficiency Virus, n: number, STI: Sexual transmitted infection, COR: Crude odds ratio, AOR: Adjusted odds ratio, CI: Confidence interval.

## Discussion

The magnitude of HIV infection among FSWs (19.9%) found in this study is high compared to the prevalence of HIV infection reported for the general population of adults in Ethiopia (1%) (2). This is due to the nature of the work which predisposes FSWs to HIV infection. The finding of this study is high compared to the pooled prevalence reported among FSWs worldwide (2.17%) (9), Latin America and the Caribbean (4.4 %), Western Europe (4.0 %), and South Asia (5.1 %) (10). Our finding is comparable to the pooled prevalence reported from America (17.3%) (3), which is high like our study; however, it is lower than the pooled prevalence reported from sub-Saharan African countries (36.9%) (11).

Moreover, the prevalence of HIV among sex workers we found in this study is higher than a report from Sudan (0.9%) (12), Afghanistan (0.19%) (13), Mexico (5.3%) (14), and Ruwanda (3.5%) (15), Our finding is low compared to HIV prevalence among sex workers reported from Uganda (38%) (16), Côte d'Ivoire (26.6%) (17), Nigeria (37.2%) (18) and South Africa (53.6%) (19). The differences observed could be due to the study period, socio-economic status of study participants, culture, and level of implementation of the prevention strategy. The comparable finding was reported from Kenya (18%) (20) and Senegal (19.8%) (21).

The finding of this study (19.9%) is lower than HIV prevalence among FSWSs reported from Ethiopia before 2014 (20–75%) (4, 6, 7) and in 2016 (24%) (22). According to a survey conducted by the Center of Disease (CDC) and Ethiopian Public Health Institute (EPHI) among FSWS in regional cities of Ethiopia between 2013 and 2014, the overall prevalence of HIV among FSWs was 23.8%, which is slightly higher than the finding of this study. Specifically, the survey reported high prevalence from Mekele city, Ethiopia (33%) and lower prevalence from Hawassa city, Ethiopia (15%) (23). The low prevalence found in this study could be due to the implementation of prevention strategies by the government and other stakeholders in the last five years which ended up in the reduction of the prevalence of HIV. However, the augmentation of HIV prevalence from 15% (23) to 19.9% may indicate a challenge in the implementation of prevention strategy and needs further investigation.

In this study, even though a significant association was not observed, the proportion of HIV infection was high among participants who did not use condoms, those with a history of STI, a history of abortion, low monthly income, and who were married. Other studies also reported similar findings (9, 12, 21).

The prevalence of HIV among FSWs at ISHDO, Hawassa was 19.9%. None of the factors assessed were significantly associated with the prevalence of HIV infections (P>0.05); however, the prevalence of HIV was higher among participants within 15–19 years age category (24.2%), who were widowed (27.3%), those with a monthly income of 501–1000 (26.9%), and those who don't use condoms (21.4%). Generally, this finding has implications as there might be a chance for the dissemination of HIV in the community and urges the importance of strengthening prevention and control.
